# Microarray Analysis of Gene Regulations and Potential Association with Acephate-Resistance and Fitness Cost in *Lygus lineolaris*

**DOI:** 10.1371/journal.pone.0037586

**Published:** 2012-05-24

**Authors:** Yu Cheng Zhu, Zibiao Guo, Yueping He, Randall Luttrell

**Affiliations:** 1 Southern Insect Management Research Unit, Agricultural Research Service, United States Department of Agriculture, Stoneville, Mississippi, United States of America; 2 State Key Laboratory Breeding Base for Zhejiang Sustainable Pest and Disease Control, Institute of Plant Protection and Microbiology, Zhejiang Academy of Agricultural Sciences, Hangzhou, Zhejiang, People's Republic of China; U. Kentucky, United States of America

## Abstract

The tarnished plant bug has become increasingly resistant to organophosphates in recent years. To better understand acephate resistance mechanisms, biological, biochemical, and molecular experiments were systematically conducted with susceptible (LLS) and acephate-selected (LLR) strains. Selection of a field population with acephate significantly increased resistance ratio to 5.9-fold, coupled with a significant increase of esterase activities by 2-fold. Microarray analysis of 6,688 genes revealed 329 up- and 333 down-regulated (≥2-fold) genes in LLR. Six esterase, three P450, and one glutathione S-transferase genes were significantly up-regulated, and no such genes were down-regulated in LLR. All vitellogenin and eggshell protein genes were significantly down-regulated in LLR. Thirteen protease genes were significantly down-regulated and only 3 were up-regulated in LLR. More than twice the number of catalysis genes and more than 3.6-fold of metabolic genes were up-regulated, respectively, as compared to those down-regulated with the same molecular and biological functions. The large portion of metabolic or catalysis genes with significant up-regulations indicated a substantial increase of metabolic detoxification in LLR. Significant increase of acephate resistance, increases of esterase activities and gene expressions, and variable esterase sequences between LLS and LLR consistently demonstrated a major esterase-mediated resistance in LLR, which was functionally provable by abolishing the resistance with esterase inhibitors. In addition, significant elevation of P450 gene expression and reduced susceptibility to imidacloprid in LLR indicated a concurrent resistance risk that may impact other classes of insecticides. This study demonstrated the first association of down-regulation of reproductive- and digestive-related genes with resistance to conventional insecticides, suggesting potential fitness costs associated with resistance development. This study shed new light on the understanding of the molecular basis of insecticide resistance, and the information is highly valuable for development of chemical control guidelines and tactics to minimize resistance and cross-resistance risks.

## Introduction

During the last decade, widespread adoptions of transgenic Bt cotton and altered chemical control schemes have allowed sucking insect populations to increase. Of these pests, the tarnished plant bug (TPB), *Lygus lineolaris*, emerged as the most economically significant [1]. Management of tarnished plant bug relies almost exclusively on chemical control. Commonly used insecticides include pyrethroids, organophosphates, carbamates, and neonicotinoids. Acephate, an organophosphorus insecticide, is among the most widely used insecticides for TPB control. In order to suppress feeding damages from tarnished plant bug and bollworm/tobacco budworm, cotton is more frequently sprayed than other major crops in the South. Over the years, tarnished plant bug has become increasingly resistant to several chemical insecticides [2], [3], [4], including acephate [5].

Acephate (Orthene) has been widely used to control tarnished plant bug on cotton in the Delta region of Mississippi, Arkansas, and Louisiana. TPB populations have developed 3- to 5-fold resistance, and control of these field populations may become difficult once resistance ratios reached 3-fold or higher [5]. Our field surveys indicate that susceptibility to acephate decreases as the growing season progresses. During late season (Aug. to Oct.), TPBs are substantially less susceptible to insecticides than those collected in May to July. Susceptibility reaches its lowest level in October. Our field surveys also indicate that TPB populations around cotton fields are less susceptible than populations around corn or soybean fields.

Another important phenomenon in studying TPB resistance to insecticides is the difficulty of maintaining resistant colonies in the laboratory. Less susceptible strains tend to have high mortality and low egg production. Although insecticide selections may increase resistance level to 10-fold or higher, selected colonies often die or fail to reproduce enough progeny for further research. Without further selection with acephate, a resistant colony can be sustained for a few generations. Resistance gradually decreased and sustained at 2- to 3-fold higher LC_50_ than susceptible strain. All of these factors limit further investigation of resistance mechanisms in this important insect pest.

With multiple generations per year, high mobility, and existence of differential activities of major detoxification enzymes [6], field populations of TPB across the Delta region have potential to evolve high levels of resistance to multiple insecticides, especially when they are under high selection pressure. Examinations of acephate resistance mechanisms have not been conducted previously, except for a few limited sequence and gene expression level comparison of one esterase and one glutathione S-transferase related to malathion-resistant TPB [7], [8]. Increased economic importance and reports of pyrethroid and organophosphate resistance [4], [9] prompted our research to better understand the mechanisms of acephate resistance in this cotton pest. By integrating biological and biochemical assays with novel microarray and other molecular analytic tools, this study compared more than seven thousand genes simultaneously in acephate-susceptible (LLS) and –selected (LLR) tarnished plant bugs. Results are expected to generate a general picture of acephate resistance in TPB and help explain how TPB evolves resistance to the chemical. In addition, this study provides insight into potential fitness costs associated with acephate resistance and possible development of multiple and cross resistance to other classes of insecticides.

## Materials and Methods

### Chemicals

The Pierce Coomassie plus protein assay kit (23238) was purchased from ThermoFisher Sci. (Pittsburgh, PA, USA). Alpha-naphthyl acetate (1-NA or α-NA) (N8505), beta-naphthyl acetate (2-NA or β-NA) (N6875), ρ-nitrophenyl acetate (PNPA) (N8130), 1-chloro-2,4-dinitrobenzene (CDNB) (23,732-9), fast blue salt (D9805), L-glutathione (GSH) reduced (G6529) were purchased from Sigma Chemical Co. (St. Louis, MO).

### Insect laboratory colony and field collection

A laboratory colony was provided by Kathy Knighten and Fred Musser at Mississippi State University. The colony has been maintained on artificial diet for seven years without exposure to insecticide. Wild adults are introduced into this colony in the spring of each year. This colony was used as a standard susceptible strain (LLS). Resistance ratios (LC_50_ of field-collected population/LC_50_ of laboratory strain) were calculated for field and laboratory strains relative to LLS. A field population (Lula) was collected in October 2010 from pigweed around a cotton field west of Lula, Mississippi. Acephate-selected bugs (Lula600) were from an acephate (Orthene® 90WP, Valent, Walnut Creek, CA) treatment of the Lula field population. Approximately 45,000 bugs were collected and selected with Orthene 90WP at 600 mg/L (or 540 mg a.i./L acephate). Adults were held in a cage (W×D×H: 60×60×60 cm) covered with 16×16 mesh metal screen on all sides except the bottom (metal sheet). Approximately 20 mL of acephate solution was used to treat the cage thoroughly from the top and all 4 sides using a hand sprayer. After treatment with Orthene (600 mg/L 90WP) for approximately 12 h, survivors were transferred into a 3.8 L container covered with a fine net cloth (10 grids/cm) and fed fresh green beans. After the first acephate treatment for 6 d, survivors were subjected to a dose-response bioassay. Six acephate concentrations were prepared at 300, 500, 750, 1,000, 1,500, and 2,000 mg/L in d-H_2_O. A water only control was also included. Three replicates were used for each concentration, and 17 adults were used for each replicate. The adults were placed in 3.8 L container and sprayed with acephate solutions. After exposure for 10 m, the adults, along with 4 fresh green bean pods, were transferred into a clean plastic container (diameter[D]×height[H]: 10.5×7 cm). Bugs were maintained at 21°C and 14∶10 (L:D) h. Mortality was recorded after 48 h and LC_50_s were calculated using SAS Probit analysis [10]. Survivors from Orthene 90WP treatment at 2,000 mg/L (LLR with resistance ratio >25-fold) were used for comparison of gene regulation using microarray analysis.

### Enzyme activity assay

Esterase and glutathione S-transferase activities were comparatively examined using the protocols described by Zhu et al. [6]. In brief, nine individual tarnished plant bugs from each sample were homogenized in sodium phosphate buffer. The homogenate was centrifuged at 10,000×g for 5 minutes at 4°C. Protein concentrations were determined using the Pierce protein assay kit which utilizes the Bradford method [11]. To determine esterase activity, micro-titer plate assays were conducted using α-NA, β-NA, and PNPA as substrates. A Bio-Tek ELx808iu plate reader (Winooski, VT) was used to monitor α-NA and β-NA reactions at 450 nm for 10 minutes with measurements taken every 15 seconds [12]. For assays with PNPA, reactions were assayed at 405 nm for 10 minutes with readings taken every 15 seconds [13]. To determine glutathione S-transferase activities, micro-titer plate assays were conducted using CDNB as substrate. The reactions were monitored at 340 nm for 10 minutes with readings taken every 15 seconds [12].

### RNA preparation, cDNA library construction and sequencing

Three adults (per tube) of *L. lineolaris* were homogenized in 1000 mL TriZol reagent (Invitrogen, Carlsbad, CA). Three to five replicates were included for each sample. mRNA was purified from total RNA using NucleoTrap mRNA purification kit (BD Bioscience Clontech, Palo Alto, CA). The Creator Smart cDNA Library Construction Kit (BD Bioscience Clontech) was used for cDNA library construction, by following manufacturer's instructions and modified protocols described by Zhu et al. [14]. Approximately 1 µg mRNA was used for reverse transcription and cDNA library construction. cDNA was ligated into pDNR-LIB vector (Clontech). The ligation was used to transform TOP10 competent cells (Invitrogen), which then were plated on chloramphenicol-agar plates. Approximately 30,000 clones were obtained and sequenced with a M13 forward primer on an ABI 3730XL sequencer (Applied Biosystems Inc., Foster City, CA) located at the Genomics and Bioinformatics Research Unit, USDA-ARS, in Stoneville, MS.

### Sequence data processing and expression gene chips

After vector trimming and assembling using DNAStar (Ver. 8, Madison, WI), sequences were subjected to a similarity search for putative identity against protein and nucleotide databases of the GenBank in the National Center for Biotechnology Information (http://blast.ncbi.nlm.nih.gov/Blast.cgi) using Blastx NR, Blastn, and tBlastX protocols of Blast2GO software (http://www.blast2go.org/) [15], [16] with 10^−3^ for cutoff *E-*value.

### Acquiring microarray data

Roche NimbleGen 72 K gene expression chips in 4-plex format (Roche NimbleGen, Inc., Madison, WI) were used to compare global gene expression between the acephate-selected (LLR) and non-selected (LLS) strains of TPB. A 60-bp specific oligonucleotide was designed and synthesized as a probe. Approximately 35,000 probes (average of 5 probes per cDNA) were synthesized and printed on each gene expression chip. Microarray analysis was processed using standard NimbleGen array protocols. Total RNA was extracted from adults using TriZol reagent (Invitrogen). Double strand cDNAs were synthesized by using the SuperScript Double-Stranded cDNA Synthesis Kit (Invitrogen) according to the manufacturer's protocols. Double strand cDNA samples were labeled with One-color DNA Labeling Kit and hybridized to the microarray chips. Microarray data were acquired according to NimbleScan v.25 User's Guide through Florida State University Microarray processing facility. Four arrays (4 replicates) of 72 K NimbleGen expression chips were processed for each sample.

### Analysis of microarray data

After gene expression data were obtained from 4×72 K array processing, ArrayStar® software (DNAStar, Inc., Madison, WI) was used to analyze and compare microarray data between LLS and LLR. Expression data were log2-transformed and normalized through quantile normalization [17], and gene calls were generated using a Robust Multichip Average (RMA) algorithm [18]. Normalized data were analyzed using classical parametric statistics. *P*-values were calculated using Modified *t*-test. Clustering techniques, including the most popular “hierarchical” clustering and “k-means” clustering, were applied in the microarray data analysis. Clustering aims at dividing data points (gene or samples) into groups (clusters) using measures of similarity, such as correlation or Eucliden distance [19]. Hierarchical clustering creates a hierarchical, tree-like structure of data, and may be displayed using a “heat map”. By using hierarchical clustering in this study, the expression levels of each gene could be quantitatively compared side by side between LLS and LLR strains. Scatter plotting was also applied to generate a distribution of more than 6,688 genes tested in an effort to locate differentially expressed genes between LLS and LLR. A fold-change cutoff of 2 and p-value threshold of 0.05 were used to determine differential gene expression (www.illumina.com/documents/products/whitepapers/whitepaper_RNASeq_to_arrays_comparison.pdf).

### Cloning full-length cDNAs coding for esterases

cDNA library sequencing yielded several clones which matched esterase cDNAs in GenBank. Cloning of full-length cDNAs was achieved using procedures similar to those of Zhu et al. [7] with some modifications as described by Yang et al. [20]. Briefly, to obtain the full length cDNAs, total RNA was extracted from adults. The SuperScript First Strand cDNA Synthesis kit (Invitrogen) was used in a reverse transcriptase polymerase chain reaction (RT-PCR) with 5 µg of total RNA and an oligo-dT primer for cDNA synthesis. Forward primers were designed and used with oligo-dT primer in RT-PCR reaction to amplify 3′-end of the esterase cDNA. The 5′-end of cDNA for each of the esterases was obtained by using the 5′ rapid amplification of cDNA end (5′ RACE) system (Invitrogen). Two to three specific reverse primers for each of the three esterases were designed and used in semi-nested amplifications with a forward abridged anchor primer from 5′-RACE kit. The 5′-end of the cDNA was isolated and C-tailed, and then cloned into a pGEM-T vector (Promega). Plasmid DNAs were prepared and sequenced using an ABI 3730XL DNA analyzer to confirm full coding sequences of the esterases. To obtain error-proof full-length cDNAs, total RNAs from LLS and LLR were used for synthesizing RT-cDNA. RT-PCR amplification was repeated with a Platinum High Fidelity Taq DNA polymerase (Invitrogen). The PCR products were purified using Qiaquick PCR purification kit (Qiagen) and sequenced from both directions as described above.

### Verification of esterase gene expressions using real-time PCR (qRT–PCR)

Differential expression of 4 up-regulated esterase genes (LLE1–4), detected by the microarray analysis, were validated by qRT-PCR according to methods described by Yang et al. [20]. TPB adults were collected in October 2011 from Lula, Mississippi, the same location of the original LLR colony. Collected bugs were divided into two groups. One group of bugs were treated with 1,000 mg/L acephate and maintained on treated green bean (21°C, L:D = 14∶10) for 7 days (Lula1000). Another group of the bugs were untreated and maintained under the same conditions for 7 days as an aging control (Lula CK). LLS (untreated) was also included as a control.

The qRT–PCR assays were performed in a 25 µl reaction volume using iScript™ One-step RT-PCR Kit with SYBR Green (Bio-Rad, Hercules, CA) in a thermal cycler PTC-200 with Chrome4 detector attached (Bio-Rad). The qRT-PCR thermal cycling profile was programmed to run at 50°C for 10 min of cDNA synthesis, 95°C for 5 min of iScript reverse transcriptase deactivation, followed by 40 cycles of 95°C for 10 s and 55°C for 30 s. A melting curve thermal cycling from 55°C to 95°C with an increment of 1°C for 10 s was added to check amplification specificity. Opticon Monitor 3 (Bio-Rad, Hercules, CA) was used to control all PCR reactions and data output. To obtain absolute quantities of each gene, two steps of qRT-PCR [20] were performed for each esterase gene. First qRT-PCR was conducted to amplify ribosomal 18S gene to estimate RNA concentration for each sample with 18S housekeeper gene as an internal standard. RNA samples were adjusted to the same concentration based on the first qRT-PCR with 18S as the internal standard. Second qRT-PCR was conducted to achieve an absolute estimation using standardized RNA samples and target gene (LLE1–4) cDNA as an internal standard. Three replications (samples) were used for each treatment, and 5 TPB adults were included in each RNA preparation. Gene expression quantity was automatically calculated by the Opticon Monitor 3. Quantity of the target gene was calculated to pg per µg of total RNA (Mean±SE).

### Examination of dose responses of field populations to acephate and imidacloprid

To detect potential resistance to multiple insecticides in TPB, dose responses to Orthene 90WP (80 mg/L or 72 mg a.i./L acephate) and imidacloprid 2 L (85 mg/L or 18.19 mg a.i./L) were evaluated in different field populations. The concentration for each insecticide was similar to the LC_50_ value (Note: this value was lower than the LC_50_ in Table 1, because both bugs and food were sprayed in this experiment) against the laboratory susceptible strain (LLS). TPBs were collected in September 2011 from northwest Mississippi. Approximately 25 bugs were used for each replicate and 3 replicates were used for each population. Bugs along with 4 green bean pods were placed in a plastic container (D×H: 10.5×7 cm). A 9-cm hole was cut on lid and covered with a fine net cloth (10 grids/cm). Five hundred µl of insecticide solution was delivered into the container using a modified Potter Spray Tower. The sprayer was set at 7.5 psi with spray distance of 30.5 cm to ensure a uniform deposition of insecticide mist on inner surface of the container, green beans, and bugs. Mortality was recorded 48 h after treatment.

**Table 1:** Biological response and enzymatic activities in acephate-treated tarnished plant bug.

Insect	Bioassay		Esterase: α-NA		Esterase: β-NA		Esterase: PNPA		GST: CDNB	
	LC_50_ a.i. (95%FL)	Ratio	MV±SE	Ratio	MV±SE	Ratio	MV±SE	Ratio	MV±SE	Ratio
LLS	148.3(122.9–174.8)	1	21.6±2	1	19.4±1.5	1	29.7±3.5	1	39.2±1.6	1
Lula*	321.1(257.8–402.5)	2.2	54.3±6.3	2.5	27.3±2.4	1.4	39.2±3.9	1.3	27.7±1.9	0.7
Lula600**	874.4(742.7–1042)	5.9	97.7±14.3	4.5	55.8±9.1	2.9	81.7±8.2	2.8	35.9±3.1	0.9

* Natural field population collected in Lula, Mississippi.

** Field population collected in Lula, Mississippi and treated with 600 mg/L of acephate (90WP) before it was used for assays.

### Examination of synergistic effect of esterase inhibitors on acephate toxicity against TPB

A field population was collected in April 2012 from Leland, MS, and treated with 72 mg a.i./L acephate. A laboratory colony (LLX) was collected in 2010 from many locations in Delta regions of Mississippi and Arkansas, and was selected with 225 mg/L acephate. In this experiment, LLX colony was treated with 144 mg/L acephate. DEF or TPP solution at 1% was prepared by dissolving inhibitor in ethanol, thereafter, an equal volume of dd-H_2_O was added to the solution. The bugs were sprayed with inhibitor solution one hour before they treated with acephate. The bugs in control were treated with 50% ethanol in water. Spray tower settings and spray volume were the same as described above. Mortality was recorded 48 h after treatment.

## Results

### Comparison of acephate-susceptibility and enzyme activities

The acephate LC_50_ for the susceptible strain (LLS) was 148.3 mg a.i./L (Table 1). The LC_50_ for the Lula field population was 2.2-fold higher than LLS. After selection with 600 mg/L Orthene 90WP, the observed LC_50_ reached 5.9-fold higher than LLS and 2.7-fold higher than the unselected Lula field population. Esterase activity determined with α-NA was 2.5-fold higher in Lula field population and 4.5-fold higher in the acephate selected bugs (Lula600) than LLS. The β-NA and PNPA activities also significantly increased by1.3–1.4-fold in Lula, and by 2.8- to 2.9-fold in Lula600, respectively (Table 1). Unlike the esterases, glutathione S-transferase activity in both Lula and Lula600 decreased by 1.4- and 1.1-fold (Table 1).

### Microarray analysis of gene regulation in LLR

#### Identification of differentially expressed genes in acephate-resistant bug (LLR)

A total of 7,446 unique contigs and singletons were obtained from cDNA library sequencing, and 6,688 genes had valid expression values from microarray analysis. Hybridization signals were analyzed and gene expression changes in LLR were presented as mean of fold changes when compared to the LLS. Of 6,688 genes examined in the LLS and LLR strains, approximately 9.9% genes were up- or down-regulated in the LLR. Expressions of the remaining 90% of genes were not significantly different between LLS and LLR. Based on *P* values (*P* <0.05) and fold change (≥2), significant differences in mRNA levels were detected in 662 genes between the LLS and LLR, which included 329 up-regulated and 333 down-regulated genes in LLR. Among the 662 differentially expressed genes, only 225 genes were putatively identified using Blast2go search of GenBank, including 107 up-regulated (Table 2) and 118 down-regulated (Table 3) genes in the LLR. Identities of the remaining 437 genes have not been determined in similarity search of GenBank using Blast2go.

**Table 2:** Identification of 107 significantly up-regulated (≥2-fold) genes in LLR using microarrays and analyzed with ArrayStar and Blat2go protocol (www.blast2go.org).

Seq ID	Sequence annotation	Fold change	P value
L1103	26s ribosomal rna boehmeria macrophylla scabrella	2.02	0.0245
L6649	acid phosphatase-1	2.90	0.0000845
L869	aggrecan core	2.09	0.000243
L3143	aldo-keto reductase	3.45	0.000687
L2988	alpha-amylase	2.58	3.24×10∧-6
L542	alpha-amylase	2.44	0.0000186
L3683	alpha-amylase 1	3.83	1.40×10∧-6
L4739	alpha-amylase 1	3.70	3.07×10∧-6
L6184	alpha-amylase 1	2.36	5.31×10∧-6
L881	antigen 5 precursor	2.10	0.00124
L6486	antigen 5 scp domain-containing	2.59	7.90×10∧-6
L186	antigen-5-like protein precursor	2.42	0.00155
L4347	carbonyl reductase	4.04	0.0000102
L5131	carbonyl reductase	5.17	3.16×10∧-6
L5648	carbonyl reductase	2.68	0.0000357
L305	carboxypeptidase b	2.31	0.0000215
L1451	carboxypeptidase b-like	2.34	0.0000213
LL_390	carboxypeptidase cpvl precursor	3.12	7.07×10∧-6
L2051	cell surface sd repeat protein precursor	254.87	0.0000377
L4783	chemosensory protein a 7a	2.34	0.000104
L2075	circumsporozoite protein	2.06	0.000731
L861	circumsporozoite protein	2.50	0.000165
L5167	counting factor associated protein d-like	2.46	0.0000303
L6543	cuticular protein lcp family 2 mrna nasonia vitripennis	3.19	0.00265
L194	cysteine proteinase 2-like digestive enzyme	2.75	0.000159
L3359	cytochrome p450	3.00	0.00168
LL_39	cytochrome p450	2.81	0.000373
L4510	cytochrome p450 6a8	2.70	0.0000229
L2414	elongation of long chain fatty acids protein aael008004	2.58	0.000146
LL_214	enth domain-containing protein	3.33	0.0000125
L1233	esterase [Lygus lineolaris]-1	2.60	0.000102
L2508	esterase-2	2.14	0.00018
L2520	esterase [Lygus lineolaris]-3	2.57	6.67×10∧-6
L5104	esterase-4	7.57	5.76×10∧-7
LL_227	carboxylesterase-6	2.70	0.000343
L6522	esterase fe4-7	2.17	0.000128
L1833	GK17235 [Drosophila willistoni]	7.23	5.14×10∧-6
L4183	GK17235 [Drosophila willistoni]	8.69	0.0000104
L6147	glutathione s-transferase	2.14	0.0000217
L5529	glycoside hydrolases	3.03	3.03×10∧-6
L2609	hemagglutinin family protein	2.79	0.0645
L3629	hypothetical protein	16.29	0.0000112
L216	hypothetical protein LOC100678289 (N. vitripennis)	2.11	0.000078
L5474	hypothetical protein LOC100679379 [N. vitripennis]	3.03	8.32×10∧-6
LL_225	hypothetical protein LOC100679379 [N. vitripennis]	2.39	0.00242
L1323	immunodominant interspersed repeat antigen	2.22	0.0000199
LL_322	immunodominant interspersed repeat antigen	2.53	0.000425
L5610	isoform a	2.16	0.0000488
L2469	isoform b	2.46	0.0000358
L501	kynurenine-oxoglutarate transaminase 3-like	2.08	9.31×10∧-6
LL_632	liprin-beta-2- partial	2.72	0.0000129
L2869	luciferin-regenerating enzyme	2.26	0.0000277
L4778	microsatellite ll-5 sequence apolygus lucorum	2.14	0.0012
L1939	mitochondrial import receptor subunit tom20 homolog	2.05	0.0122
LL_248	mus musculus bac clone rp24-358i7 from chromosome	2.25	0.0000311
L3451	mus musculus chromosome clone rp23-	3.54	2.74×10∧-6
L4498	mus musculus chromosome clone rp23-	2.81	0.000247
L1449	mus musculus chromosome clone rp23-	2.26	0.00342
LL_135	mycobacterium complete genome	2.18	0.00135
L753	neuroparsin 1 precursor	3.87	3.87×10∧-6
L4114	nocardia farcinica ifm 10152 complete genome	2.32	0.00041
LL_574	nucleolar protein family 2 (h aca small nucleolar rnps)	2.40	0.0000937
L2338	omega-amidase nit2-like	42.92	9.60×10∧-7
L4563	pancreatic lipase-related protein 2-like	2.40	0.0000264
L2487	pancreatic triacylglycerol lipase	3.38	0.0000234
L380	partial genome (lygus lineolaris)	2.54	0.386
L964	partial genome (lygus lineolaris)	2.15	0.527
L4630	peptidyl-prolyl cis-trans isomerase 10	2.80	4.80×10∧-6
L5883	phospholipid scramblase 1	2.06	0.00206
L4906	plasmodium falciparum 3d7 chromosome 8	4.07	0.00218
L1904	endo-polygalacturonase b	2.16	0.000925
L1124	polygalacturonase 4	2.27	3.32×10∧-6
L6079	polygalacturonase	3.65	8.55×10∧-6
L3578	endopolygalacturonase	5.61	0.0000182
L3333	polygalacturonase	4.11	0.000803
L518	polygalacturonase pg1	2.02	0.0000231
L4832	endopolygalacturonase	13.92	8.72×10∧-6
L2316	endo-polygalacturonase	2.64	0.0000782
L4070	predicted protein [Nematostella vectensis]	2.17	0.000601
L1882	Predicted: C09D4.2 [Hydra magnipapillata]	2.32	0.0000533
L600	Predicted: ENSANGP00000004103 [S. purpuratus]	3.11	0.0000214
L28	protein CLONEX_01831 ZP_03289628	2.40	0.434
L6646	pyridoxamine 5 -phosphate oxidase	2.08	0.00456
L2427	regucalcin	2.26	0.0000378
L2931	regucalcin	2.21	0.000119
L4962	secreted salivary gland	3.80	0.0000262
L5724	serine 3-dehydrogenase	2.13	0.0000213
LL_55	serine protease	36.71	0.000308
L4164	serine protease inhibitor serpin-4	2.99	0.0000112
L3882	serine proteinase stubble	3.92	0.000638
L5938	sp185 333	2.28	0.000225
L5880	spore coat assembly protein	3.37	0.000209
LL_773	spore coat assembly protein exsa	2.92	6.57×10∧-6
L1658	st14a protein	2.29	0.0645
L5575	tetraodon nigroviridis full-length cdna	2.10	0.0032
L5521	thaumatin-like protein	2.31	0.0000111
L6464	thaumatin-like protein	2.01	0.0000163
L3539	transmembrane protease	30.46	0.000386
L2022	trypsin: salivary	3.52	2.19×10∧-6
L3508	venom serine carboxypeptidase-like	5.36	2.48×10∧-6
L4429	venom serine carboxypeptidase-like	2.14	0.000131
L2932	ves g 5 allergen	2.39	0.000061
L4213	ves g 5 allergen	2.85	0.000014
LL_466	ves g 5 allergen	2.24	0.0000212
L868	vitellogenic carboxypeptidase	2.26	0.0000582
L2069	xenopus tropicalis clone ch216- complete sequence	3.38	0.0153
L4505	zinc metalloproteinase c	2.98	0.0000355

**Table 3:** Identification of 118 significantly down-regulated gene (≥2-fold) in LLR using microarrays and analyzed with ArrayStar and Blat2go protocol (www.blast2go.org).

Seq ID	Sequence annotation	Fold change	P value
L2460	acyrthosiphon pisum protein takeout-like mrna	2.04	0.00091
L4322	alkylated dna repair protein alkb-like protein 8	3.38	0.000356
LL_476	apolygus lucorum microsatellite ll-5 sequence	2.30	0.00369
L6435	atp-binding cassette superfamily	4.49	0.00267
L6338	ax4 myb domain-containing protein cds	4.00	0.0000124
L4040	brachyspira intermedia pws complete genome	2.18	0.0116
L2273	callithrix jacchus sonic hedgehog mrna	3.06	0.00976
L5702	candidatus pelagibacter complete genome	2.02	0.0184
L1023	cathepsin a	3.46	3.07×10∧-6
L3275	cathepsin a	2.35	3.04×10∧-6
L3761	cathepsin a	2.61	0.0000398
L4973	cathepsin a	2.82	0.0000325
L6192	cathepsin a	2.72	3.49×10∧-6
L6305	cathepsin b precursor	2.32	0.000194
L6308	cathepsin d	2.48	0.000595
L6180	cathepsin l	15.51	4.35×10∧-7
LL_76	cathepsin l	2.18	6.58×10∧-6
L3298	cathepsin l precursor (major excreted protein) mrna	2.57	0.0000781
L4110	cathepsin r-like	2.15	0.000068
L496	receptor for egg jelly 2 protein precursor	17.67	6.25×10∧-7
L2462	hypothetical protein	71.20	2.69×10∧-6
L2548	chromosome 3 clone RP11-556G18 map 3p (H. sapiens)	8.81	4.11×10∧-7
L5687	chromosome clone rp11- (homo sapiens)	4.62	0.0000177
L1965	chromosome clone rp23- (mus musculus)	2.07	0.000113
L3117	chromosome clone rp23- (mus musculus)	100.39	0.0000318
L5568	chromosome clone rp23- (mus musculus)	15.46	5.17×10∧-6
L1035	conserved protein	38.71	1.60×10∧-7
L5626	conserved protein	29.51	6.99×10∧-7
LL_395	conserved protein	48.71	6.21×10∧-7
L3444	counting factor associated protein d-like	2.49	0.0000287
L2459	cuticle protein 6	2.08	0.0244
L787	cuticular protein 62bc	4.62	0.00108
L3905	cysteine protease cp5	2.60	6.57×10∧-6
L561	cytoplasmic polyadenylation element-binding protein 2	27.52	1.72×10∧-6
LL_46	cytoplasmic polyadenylation element-binding protein 2	28.05	1.06×10∧-6
L583	defensin a	4.22	1.08×10∧-6
L562	drosophila erecta gg13471 (dere\gg13471) mrna	2.56	0.00976
L1412	drosophila mojavensis gi24712 (dmoj\gi24712) mrna	12.38	1.66×10∧-7
L1974	drosophila mojavensis gi24712 (dmoj\gi24712) mrna	16.90	5.64×10∧-7
L5888	eggshell RP45 [Rhodnius prolixus]	19.78	1.89×10∧-7
L930	eggshell rhodnius prolixus rp45 partial cds	7.56	1.60×10∧-7
L4775	eggshell RP45 [Rhodnius prolixus]	147.45	0.0000252
L3689	endopolygalacturonase	5.88	5.21×10∧-6
L410	eukaryotic aspartyl protease family protein	2.66	0.000479
L4254	follicle cell protein 3c-1	10.72	0.000117
LL_358	foot protein 1 variant 4	7.11	1.86×10∧-7
L755	ga24146 (dpse\ga24146) mrna	25.37	5.29×10∧-6
L6045	gametocyte-specific factor 1	2.26	0.00597
LL_304	gamma-interferon-inducible lysosomal thiol reductase	5.41	0.0000419
L4147	genomic chromosome clone: (lotus japonicus)	19.29	0.000014
L552	granzyme h-like	3.62	0.0062
L1938	gryllus bimaculatus gbcontig30355	5.31	0.0000247
L6235	gtp-binding protein alpha gna	2.57	0.00257
LL_573	heinz 1706 chromosome 1 clone hba-57j16 map equence	4.13	0.0000213
L4603	histone h1	2.42	0.000541
L369	homeobox protein pknox2 isoform 2	2.27	0.000135
L5329	hypothetical protein [C. tropicalis MYA-3404]	2.02	0.00295
L5570	isoform a	2.07	0.000726
L1141	keratin-associated protein 10-4-like mrna	5.33	3.00×10∧-6
L5833	laminin subunit alpha	2.08	0.0000552
L1954	lipase 1 precursor	2.48	0.000117
L6274	lipase 3	3.10	0.00138
L2994	lysosomal acid	2.81	0.000292
L5540	MAA_09280 [Metarhizium anisopliae ARSEF 23]	2.50	0.041
L3946	merozoite surface protein	152.53	0.0000356
L3911	mitotic spindle assembly checkpoint protein mad2	2.07	0.000304
L1931	multidrug resistance protein 1	2.15	0.000514
L261	nasonia vitripennis protein piwi-like mrna	2.32	0.00215
L6396	nematostella vectensis protein partial mrna	7.02	3.24×10∧-7
L3125	odorant-binding protein 5	2.07	0.259
L3878	oryza sativa japonica group os04g0107700 complete cds	2.49	4.64×10∧-6
L3316	ov09f09 dna polymerase delta subunit 3 complete cds	4.22	0.0000434
LL_617	pao retrotransposon peptidase family protein	2.09	0.000151
LL_645	pediculus humanus corporis protein takeout mrna	2.87	0.000225
L4935	peptidase c1a papain	5.77	6.36×10∧-7
L871	peptide-n4-(n-acetyl-β-d-glucosaminyl) asparaginase amidase	65.26	0.0000227
L1761	predicted protein [Nematostella vectensis]	4.62	0.00192
L3170	prolixicin antimicrobial peptide	3.28	1.50×10∧-6
L1206	pseudonocardia dioxanivorans complete genome	3.44	3.54×10∧-6
L5409	pseudonocardia dioxanivorans complete genome	3.57	3.63×10∧-6
L6148	pseudonocardia dioxanivorans complete genome	3.82	3.04×10∧-6
LL_744	reverse ribonuclease integrase	2.12	0.000185
L2434	ribosomal protein l27e	2.18	0.000763
L6457	schistosoma mansoni strain puerto rico chromosome	2.68	0.00563
L4730	secreted salivary gland	2.80	4.17×10∧-6
LL_19	secreted salivary gland mrna (ixodes scapularis)	33.51	0.0000169
LL_20	secreted salivary gland mrna (ixodes scapularis)	27.27	6.85×10∧-7
L605	seminal fluid protein hacp037	2.01	0.0985
LL_54	seminal fluid protein hacp037	2.18	0.00268
L3098	serine carboxypeptidase-like enzyme	11.01	0.000138
L1017	serine protease	34.41	6.20×10∧-7
L5977	serine protease	146.15	0.0000199
L6207	serine protease	10.29	0.0000794
LL_104	serine protease	3.44	7.25×10∧-6
LL_119	serine protease	30.08	6.11×10∧-7
LL_672	serine protease	2.42	7.21×10∧-6
L3307	serine protease [Creontiades dilutus]	2.12	0.0000554
L772	serine protease inhibitor 3-like	2.09	0.000336
LL_207	serine protease inhibitor 3-like	2.18	0.000211
L4053	serine protease nudel	4.92	0.0000199
LL_417	serine protease nudel	6.70	0.0000291
L5464	serrano protein	4.43	0.000478
L3469	srz2 chromosome 20 complete dna sequence	6.88	1.73×10∧-6
L2301	strongylocentrotus purpuratus mgc83166 protein mrna	2.09	0.0396
L3344	subfamily c1a unassigned peptidase (c01 family)	2.76	0.0000129
L171	suppression of tumorigenicity 14 (colon carcinoma) b	2.23	0.0000565
L5341	talinum paniculatum 26s ribosomal rna partial sequence	8.39	5.76×10∧-6
L5396	tpa: cuticle protein	7.04	0.000362
L6645	translation initiation factor if-2	25.74	0.0000226
L677	tribolium castaneum ga10301-pa mrna	24.39	0.00019
L544	unknown [Lygus lineolaris]	2.12	0.0000113
L5739	venom serine carboxypeptidase-like	2.49	0.000475
L1935	ves g 5 allergen	8.68	6.26×10∧-7
L140	vitellogenin	3.34	0.000159
L4564	vitellogenin	6.26	1.99×10∧-6
LL_168	vitellogenin	3.71	2.94×10∧-6
L3867	zebrafish ch211-254e10 in linkage group complete sequence	3.90	0.000201
L2628	zinc finger c4h2 domain-containing transcript variant 2 mrna	2.37	0.0557

#### Comparison of gene expression levels by hierarchical clustering

**Figure 1:**
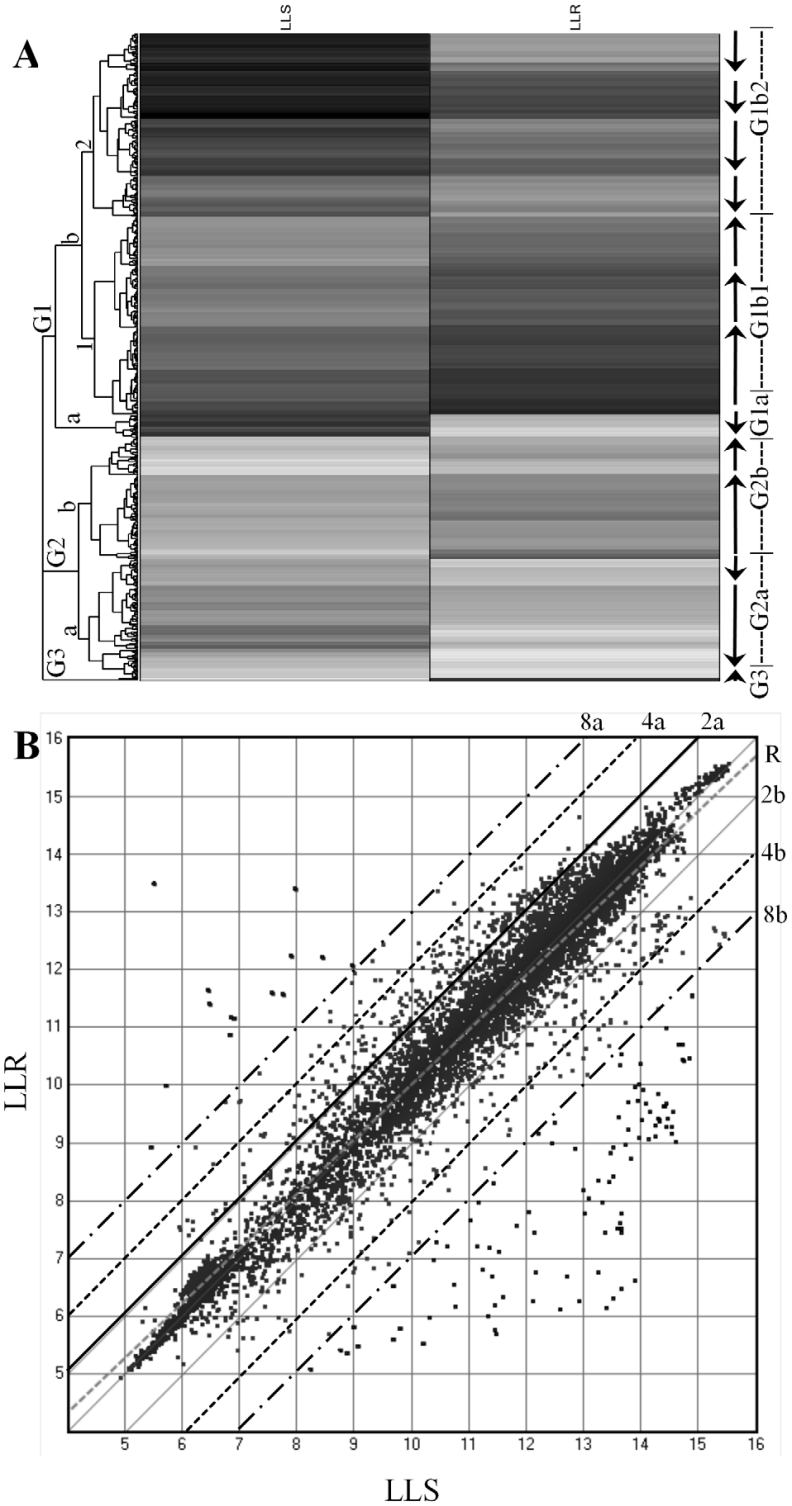
Analysis of microarray data and comparison of 6,688 gene expression levels between acephate-susceptible (LLS) and –resistant (LLR) tarnished plant bugs using ArrayStar software. **A**: Hierarchical clustering analysis of 329 up-regulated and 333 down-regulated genes (≥2-fold, *P*<0.05) in the LLR. Each column represented a sample (LLS or LLR) and each row represented a gene. The gray scale depicted the relative levels of gene expressions from low to high with corresponding grayscale from light (for lower expression) to dark (for higher levels). **B**: Scatter-plot comparison of 6,688 gene expression levels between LLS and LLR. The dots of the scatter plot in the upper left corner represented up-regulated genes, and the dots in low right corner represented down-regulated genes. Dots above line 2a and below line 2b represent up- and down-regulated genes by 2-fold; Dots above line 4a and below line 4b represent up- and down-regulated genes by 4-fold; Dots above line 8a and below line 8b represent up- and down-regulated genes by 8-fold.

The 329 up-regulated genes and 333 down-regulated genes (identified with microarray and Blast2go similarity search) in the LLR strain, selected based on the *P* value (*P*<0.05) and fold change (≥2), were analyzed by using hierarchical clustering analysis and plotted as a heat map with the ArrayStar software (Fig. 1A). Each column represented a sample (LLS or LLR) and each row represented a gene. The gray scale depicted relative levels of gene expressions from low to high with corresponding grayscale from light (for lower expression) to dark (for higher levels). The gene clustering at the left of the heatmap indicated the presence of three groups of genes with distinct expression profiles of 662 genes (≥2-fold change) across samples. Group 1 (G1) (413 genes) was separated into subgroups of G1a and G1b. All genes in G1a (23 genes) were down-regulated in LLR strain. G1b was separated into G1b1 (202 genes) and G1b2 (188 genes). The genes in G1b1 appeared to be up-regulated, while the genes in G1b2 were down-regulated in LLR (Fig. 1A). Group 2 had subgroups G2a (122 genes) and G2b (125 genes). Genes in G2a appeared to be down-regulated. Genes in G2b were up-regulated in LLR. Group 3 (G3) was a small group (2 genes), and the genes in G3 were highly up-regulated in the LLR strain.

#### Scatter-plot comparison of gene expression levels between LLS and LLR

A total of 6,688 valid gene expression data (log2) points (dots) of LLR were pairwise plotted against the same genes of LLS (Fig. 1B). The two sets of gene expression data showed linear correlation with R^2^ 0.89. When the Student's t-test was selected, there were 1,638 and 2,534 genes that showed significantly different between LLS and LLR at 99% and 95% confidence interval levels, respectively. Dots of the scatter plot in the upper left corner represented up-regulated genes, and dots in low right corner represented down-regulated genes (Fig. 1B). Distance between a dot and the regression line (R) indicated a gene expression level with longer distance for higher up- or down-regulation. A total of 662 genes (dots above line 2a and below line 2b) showed 2-fold changes, 191 genes (dots above line 4a and below line 4b) showed 4-fold changes, and 113 genes (dots above line 8a and below line 8b) exhibited 8-fold changes (Fig. 1B) between LLS and LLR.

#### Annotation and functional analysis of up- and down-regulated genes

**Figure 2:**
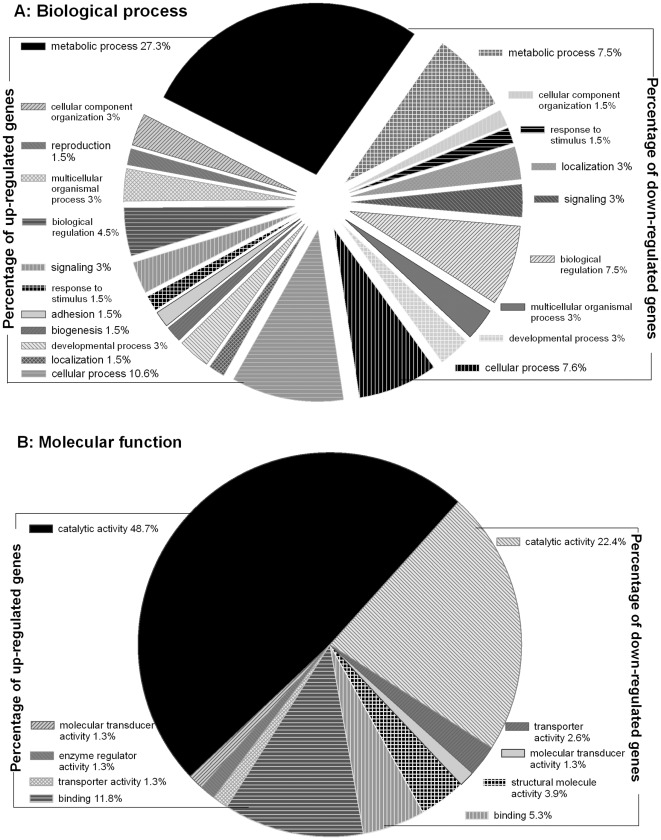
Annotation and functional analysis of 329 up-regulated and 333 down-regulated genes (≥2-fold) in the LLR. **A**: Proportion of up-regulated genes (left side) and down-regulated genes (right side) categorized based on their involvement in biological process at GO level 2 (Blast2go). **B**: Proportion of up-regulated genes (left side) and down-regulated genes (right side) categorized based on different molecular function at GO level 2 (Blast2go).

Three hundred and twenty-nine up-regulated and 333 down-regulated (≥2-fold) gene cDNAs were subjected separately to blast2go mapping and annotation to determine each putative protein's role in biological process and molecular function. Annotation with blast2go showed that 66 genes of the 662 differentially expressed genes were involved in biological processes at GO level 2, including 41 up-regulated and 25 down-regulated genes in LLR. Among the 41 up-regulated genes, 18 (27.3% of the 66 biological process-related genes) genes were associated with metabolic processes (Fig. 2A), while only 5 of the 25 (7.6% of the 66 genes) down-regulated genes were associated with the same function of metabolic process (Fig. 2A). Among the 662 differentially expressed genes, 76 genes were involved in molecular functions, including 49 up-regulated and 27 down-regulated genes at GO level 2 in LLR. Among the 49 up-regulated genes, 37 (48.7% of 76 molecular function-related genes) genes were associated with catalytic activities (Fig. 2B), while only 17 of the 27 (22.4% of the 76 genes) down-regulated genes were associated with catalytic activities. Results indicated that more than 3.6-fold of the metabolic-related genes were up-regulated compared to those down-regulated genes with the same biological function. The results also indicated a substantial increase of metabolic activities in LLR. Similarly, analysis of molecular function revealed a large portion of catalytic-related genes with significant up-regulations, suggesting an increase of catalytic activities in LLR compared to LLS.

### Potential association of up-regulated genes with altered acephate-resistance in *L. lineolaris*

#### Up-regulation of esterase genes and metabolic resistance development

**Figure 3:**
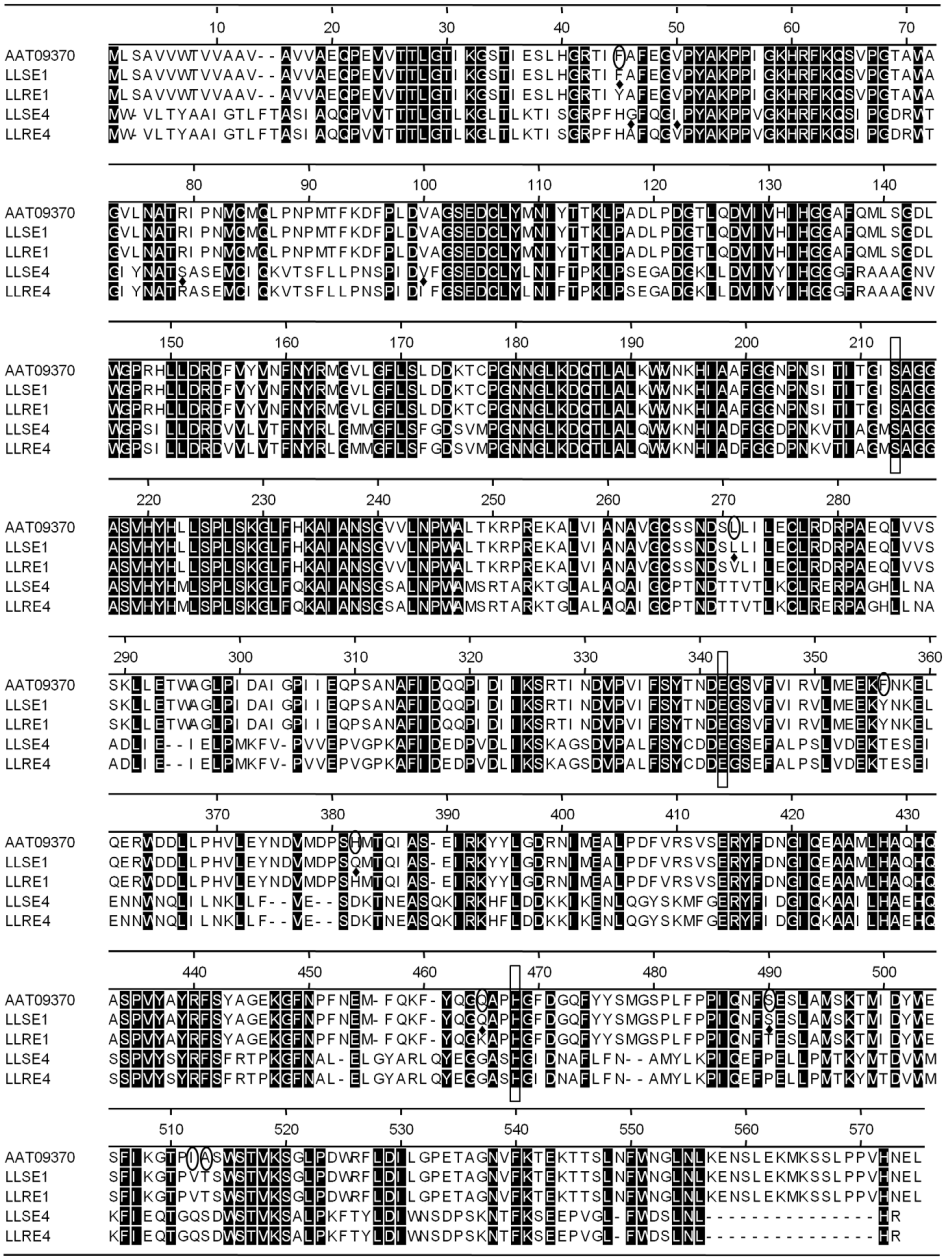
Predicted amino acid sequence of two new esterases from LLS (LLSE1 and LLSE4) and LLR (LLRE1 and LLRE4) aligned with a previously reported esterase (AAT09370) from *L. lineolaris* using Clustal W method (gap penalty: 3.0, gap length penalty: 0.2) of DNAStar MegAlign (Ver. 8). GenBank accession: LLSE1: JQ964230; LLRE1: JQ964231; LLSE4: JQ964232; LLRE4: JQ964233. Three catalytic center residues (S^213^, E^342^, and H^468^) were boxed. Amino acid substitutions between LLS and LLR are marked with ♦; Amino acid substitutions between AAT09370 and LLE1 are marked with 0. Hyphens represent sequence alignment gaps. Identical residues among all esterases are shaded with black background.

Increase of esterase activities in LLR prompted further examination and comparison of esterase gene expressions and cDNA sequences between LLS and LLR. Six esterase genes in LLR showed significantly higher gene expression levels (2.14–7.57-fold increase) than those in LLS (Table 2). No esterase genes were down-regulated in LLR (Table 3). By using cDNA library sequencing and RT-PCR, two different full length esterase cDNAs (LLE1 and LLE4) were obtained from both LLS (designated as LLSE1 and LLSE4) and LLR (designated as LLRE1 and LLRE4). LLE1 represented the most abundant esterase gene transcripts of 4 highly similar variants (data not shown) among the 6 up-regulated esterase genes in LLR. The others had one or two variants. The LLE1 showed 2.6-fold increase of the esterase gene expressions in LLR. LLE4 showed the highest increase of esterase gene expressions (7.57-fold) in LLR. Sequence alignment of deduced proteins, including a previously published esterase AAT09370 [7], revealed three conserved catalytic center residues, S^213^, E^342^, and H^468^ (Fig. 3). Searching with InterPro Scan and MyHits (http://www.expasy.ch/tools/) indicated that all putative proteins belonged to type-B carboxylesterase with conserved sequence pattern (F-[GR]-G-x(4)-[LIVM]-x-[LIV]-x-G-x-S-[STAG]-G) around serine active site at positions 200–215 (Fig. 3). The 1171-bp LLSE1 and LLRE1 had 9 nucleotide substitutions, resulting in 5 amino acid differences in the deduced 570-residue proteins between LLS and LLR. The second 1801-bp esterase cDNA, LLSE4 from LLS and LLRE4 from LLR, encoded 546-residue protein. There were 11 nucleotide and 4 amino acid substitutions between LLSE4 and LLRE4. Pair-wise sequence alignment showed that esterase sequence identities were approximately 99.1%, 52%, and 52% between AAT09370 and LLE1, AAT09370 and LLE4, and LLE1 and LLE4, respectively. The LLE1 is very similar to AAT09370, but they differ at 8 amino acid positions (Fig. 3).

#### Validation of up-regulated esterase genes using real-time PCR (qRT-PCR)

**Figure 4:**
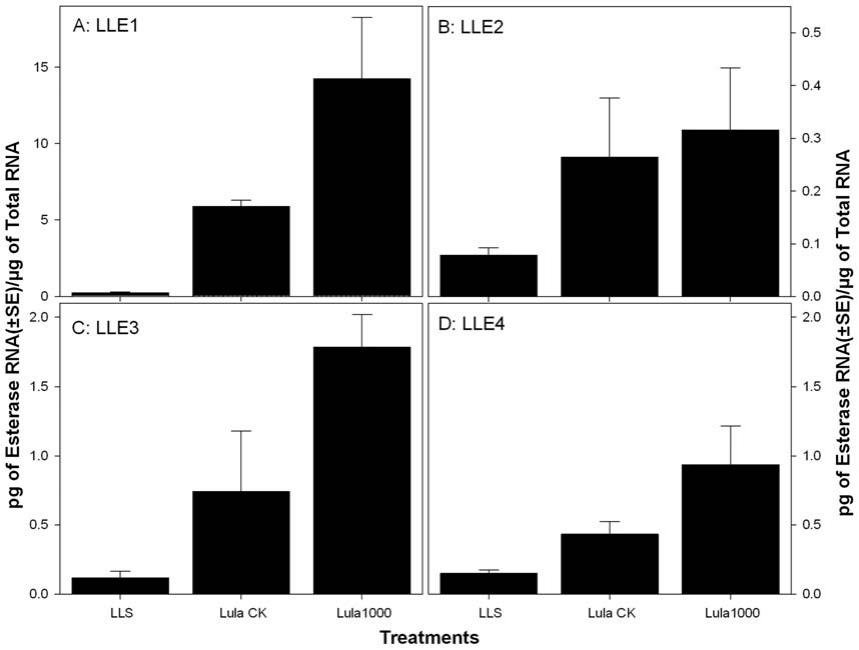
Comparison and verification of transcriptional levels of two up-regulated (detected by microarray) esterase genes (LLE1 and LLE4) using absolute estimating method in real-time PCR. LLS: laboratory susceptible strain; Lula CK: field population collected from Lula MS as an aging control; Lula1000: field population collected from Lula MS and selected with 1,000 mg/L acephate (90WP).

Real-time PCR was conducted to verify reproducibility of microarray data. Four representative genes (LLE1–4) were selected and subjected to real-time qRT-PCR validation in acephate-selected TPB adults (Lula1000) collected from the same location in 2011 as was done for LLR in 2010. Expression levels of LLE1 in Lula-CK and Lula1000 were significantly up-regulated by 24- and 57.9-fold compared to the gene expression in LLS (Fig. 4A). LLE2 gene expressions in Lula-CK and Lula1000 were also up-regulated by 3.4- and 4-fold over that of LLS (Fig. 4B). Lula-CK and Lula1000 showed 6.3- and 15-fold higher LLE3 gene expressions than LLS (Fig. 4C). LLE4 gene expressions in Lula-CK and Lula1000 were also up-regulated by 2.9- and 6.3-fold over that of LLS (Fig. 4D). Lula1000 and Lula-CK were collected from the same location and were the same age. After acephate (1,000 mg/L) selection and removal of susceptible bugs, all four esterase gene (LLE1–4) expressions increased 2.4-, 1.2-, 2.4-, and 2.2-fold, respectively. These results suggest up-regulated esterase genes are closely associated with reduced susceptibility in acephate-selected TPB (Lula600, Table 1).

#### Synergistic effect of esterase inhibitors on acephate toxicity against two TPB colonies

**Figure 5:**
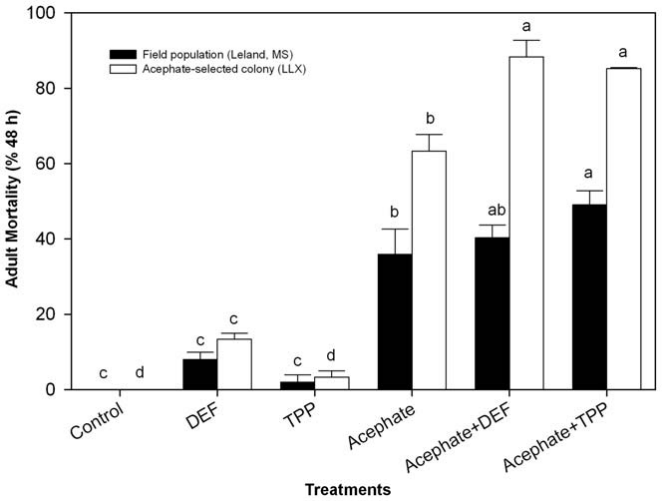
Synergistic effect of two esterase inhibitors, S,S,S-tributylphosphorotrithioate (DEF) and triphenyl phosphate (TPP) on acephate toxicity against a field population and an acephate-selected colony (LLX) of *L. lineolaris*. DEF and TPP were used at 1%; Field population from Leland was treated with acephate at 72 mg/L and acephate-selected colony (LLX) was treated with acephate at 144 mg/L.

By applying esterase inhibitor, the toxicity of acephate was increased against both field and acephate-selected colonies of TPB (Fig. 5). Approximately 35.90% of Leland TPBs were killed by 72 mg/L acephate-only treatment. DEF-only killed 7.97% of the bugs. Acephate+DEF killed 40.31% of the bugs, which was numerically higher but not significantly different from acephate-only treatment. Acephate+TPP killed 49.04% of Leland field bugs, which was significantly higher than that of acephate-only treatment. After corrections with each inhibitor's mortality, synergistic ratios of DEF and TPP were 0.98 and 1.34, respectively. Similarly, approximately 63.330% of acephate-selected TPBs (LLX) were killed by 144 mg/L acephate-only treatment. DEF-only killed 13.33% of the bugs. Acephate+DEF killed 88.33% of the bugs, which was significantly different from acephate-only treatment. Acephate+TPP killed 85.24% of Leland field bugs, which was also significantly higher than that of acephate-only treatment. After corrections with each inhibitor's mortality, synergistic ratios of DEF and TPP against LLX colony were 1.37 and 1.34, respectively.

#### Up-regulation of a glutathione S-transferase (GST) gene in LLR

By cDNA library sequencing, 19 GST cDNAs were obtained. These GST cDNAs were re-assembled into 11 highly different contigs or singletons. Multiple sequence alignment showed that average sequence identities were 30% (10–86%) among 11 predicted GST protein sequences. These GSTs had 18.6% (17–45.5%) average sequence identities with previously reported GST from *L. lineolaris* (ABC46449; [8]). Based on their relatively low sequence identities, we suggest that all 11 GSTs from this study are newly cloned GSTs. Microarray data indicated that only one GST gene (L6147) was up-regulated by 2.14-fold in LLR (Table 2). However, no GST genes were down-regulated in LLR. The 691-bp full-length cDNA encoded a 203-residue GST protein. Pair-wise alignment of protein sequences showed only 19.2% amino acid identity between this up-regulated GST in LLR and the only GST reported previously [8], suggesting a new GST might be related to reduced acephate susceptibility in *L. lineolaris*.

#### Up-regulation of Cytochrome P450 monooxygenase (P450) genes in LLR

A total of 13 partial cDNAs of P450s were obtained from cDNA library sequencing. Microarray data showed that three P450 genes were up-regulated by 2.7- to 3-fold in LLR (Table 2). No P450 genes were down-regulated in LLR (Table 3). L3359 belongs to CYP 6 family, which is commonly associated with pesticide resistance in insects. LL_39 and L4510 genes are also similar to P450s in the CYP 6 family. Comparison of 74-residue peptide close to C-termini revealed 31.9–52.8% amino acid identities among the three putative P450 proteins. Showing low sequence identities (38.9–41.7%) to 3 highly similar P450s (CYP6X1v1-3; [21]) of *L. lineolaris* in GenBank, L3359, LL_39, and L4510 may be newly sequenced P450s with potential association with insecticide resistance.

**Figure 6:**
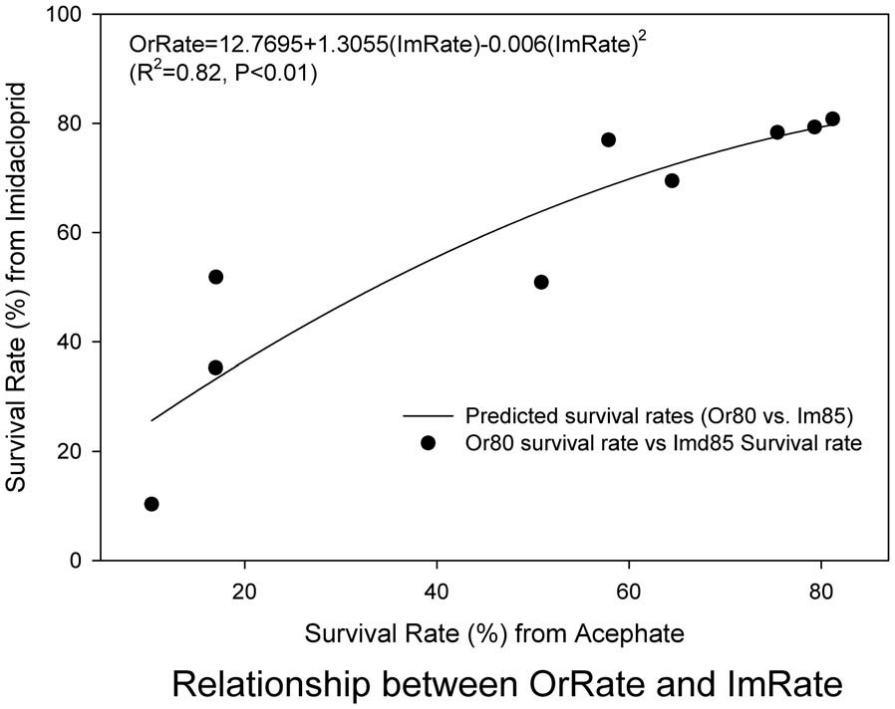
Correlation of survival rates between acephate-treated (OrRate) and imidacloprid-treated (ImRate) *L. lineolaris* collected in September, 2011 in northwest Mississippi.

To test whether over-expression of P450 genes confer multiple resistances to different insecticide classes, dose responses to acephate and imidacloprid were evaluated in 9 field populations collected from northwest Mississippi, including Lula. Results showed variable survival rates in different populations (Fig. 6). Responses to both insecticides appeared to be correlated. The population showing low susceptibility to acephate also had low susceptibility to imidacloprid. The correlation was significant with R^2^ value 0.81 (Fig. 6).

### Potential association of down-regulated genes with reduced acephate susceptibility in *L. lineolaris*

#### Down-regulation of eggshell and vitellogenin genes associated with egg production

Microarray data showed that three eggshell protein genes were down-regulated by 7.56- to 147.45-fold in LLR. In addition, three vitellogenin genes were also down-regulated by 3.34- to 6.26-fold in LLR (Table 3). Consistently, no eggshell or vitellogenin genes expressed significant increases in their gene transcript abundances in LLR (Table 2). Vitellogenin coding region of L4564 and LL_168 was overlapped with that of L140, but their cDNA sequence had only 33% and 45% identity, respectively, with L140. Therefore, L4564 and LL_168 are different vitellogenin cDNAs from L140. Because L4564 and LL_168 were not overlapping, whether they are from the same vitellogenin gene needs to be determined in a future study.

#### Down-regulation of protease and cathepsin genes associated with protein hydrolyzation

In LLR, only two protease and one salivary trypsin were up-regulated by 30.46–36.71- and 3.52-fold (Table 2), respectively. Thirteen serine protease genes showed 2.09- to 146.15-fold decrease in expression in LLR (Table 3). Another 11 cathepsin genes were also down-regulated by 2.15- to 15.51-fold in LLR (Table 3).

## Discussion

### Up-regulation of esterase genes associated with increased detoxification

Possessing ester bonds is a common feature in organophosphate insecticides, including acephate. Esterases detoxify organophosphate insecticides directly by splitting esters into an acid and an alcohol [22]. In addition, esterases might be involved in pyrethroid resistance development [23]. In this study, we consistently demonstrated that esterases are major detoxification enzymes and are responsible for the metabolic resistance closely associated with increased acephate resistance in the tarnished plant bug. By using acephate selection, we first demonstrated that elevated resistance level was closely correlated with increased esterase activities (Table 1). A previous study showed that fluctuation of esterase activities was well synchronized with seasonal increases of resistance levels in TPB [7]. Increased esterase activity in the late season likely resulted from pesticide applications on cotton. Without further exposure to insecticides in winter, the populations become less resistant. Therefore, insecticide resistance levels are closely associated with the intensity of chemical sprays. Seasonal variability in insecticide resistance are well documented [24], [25], [26]. Insecticides kill susceptible individuals and resistance levels evolve as resistant individuals increase over the growing season [27], [28].

Higher esterase activities might result from altered gene sequence and hence the protein sequence difference between LLS and LLR. Careful examination of amino acid substitutions between LLSE1 and LLRE1 revealed 5 amino acid substitutions, but these substituted amino acids were strongly similar. Besides 2 strongly similar residue substitutions, LLRE4 had one weakly similar amino acid substitution (G to A) and a different amino acid substitution (S to R), suggesting a quality change of the LLRE4 esterase. Esterases are often found in multigene families [29], [30] and it is very likely that a complex of esterases is present in the tarnished plant bug. cDNA library sequencing and microarray analysis in this study revealed multiple esterase genes and differential expressed esterase genes in LLR. Overproduction of gene transcripts might have resulted from the transcription of a single copy of the esterase gene [31] or multiple copies of the esterase genes [32], [33]. It is possible that resistant TPBs may have additional copies of esterase genes. A total of 14 esterase cDNA fragments were obtained in this study. Further assembling of these esterase cDNAs obtained at least 7 highly different esterase cDNAs or genes in the tarnished plant bug. Of the esterase genes examined, six of them were significantly up-regulated and none were down-regulated in LLR.

Synergists are often used as a diagnostic tool to characterize resistance mechanisms in pest populations. S,S,S-Tributyl phosphorotrithioate (DEF) inhibits hydrolysis of insecticides with ester linkages [34], [35]. Triphenyl phosphate (TPP) was recognized as a specific inhibitor of carboxylesterase, which synergized the effect of malathion by blocking the production of malathion mono-acid in a resistant strain of *Tribolium castaneum* [36]. Based on our 2010 data, Leland population had relatively lower resistance ratio (1.6) to acephate. In this study, DEF was unable to synergize acephate toxicity, indicating that the population had no elevated DEF-suppressible esterases. The acephate-selected LLX colony is a mixture of several populations collected from Delta regions of Mississippi and Arkansas. It maintains 2–3-fold resistance ratio relatively to the LLS colony. Significant DEF synergism to acephate in acephate-selected LLX colony indicated that elevated esterase gene expressions exist in LLX for detoxifying acephate. Unlike DEF, TPP synergized acephate toxicity in both Leland field population and acephate-selected LLX colony, suggesting that both colonies had elevated carboxylesterases. These synergistic data functionally proved that esterase-mediated metabolic detoxification is potential resistance mechanism in acephate-resistant TPB. To knock down detoxification genes, RNAi will be conducted in future study to identify specific esterase genes that are the most responsible for acephate resistance in TPB.

### Up-regulation of a glutathione S-transferase gene and potential association with metabolic detoxification in LLR

Glutathione S-transferases (GSTs) catalyze transformations of a wide range of endogenous and xenobiotic compounds, including carcinogens, therapeutic drugs, products of oxidative stress, herbicides, and insecticides [37], [38], [39]. They have the capacity to conjugate reduced glutathione on the thiol of cysteine to various electrophiles and to bind with high affinity to various hydrophobic compounds [40]. Elevated GST activity has been associated with resistance to all the major classes of insecticides, commonly through increases in transcriptional rate, rather than qualitative changes in individual enzymes [41], [42]. Of the 19 GSTs analyzed using microarray in this study, only one GST gene showed significantly higher transcripts in LLR. However, no GST was significantly down-regulated in LLR. GST enzyme activity data were also consistent with GST gene expression data. Significantly lower GST activity was found in the Lula natural population, suggesting a lower GST activity baseline than LLS (Table 1). Selection with Orthene 600 mg/L removed approximately 80% susceptible bugs from the sample causing GST activity to increase to a level similar to that of LLS. Considering GST enzyme activity, expression fold changes, and the number of genes involved, we postulate that GSTs may play a less significant role than esterases in reduced susceptibility of *L. lineolaris* to acephate.

### P450 oxidation and potential multiple resistance and cross resistance to different classes of insecticides in LLR

Cytochrome P450 enzymes (mixed function oxidases, cytochrome P450 monooxygenases, CYP), are a large and diverse class of enzymes found in virtually all insect tissues. The function of most P450 enzymes includes catalyzing the oxidation of organic substances to fulfill many important tasks, from the synthesis, degradation, and metabolic intermediations of lipids, ecdysteroids and juvenile hormones to the metabolism of xenobiotic substances of natural or synthetic origin [43]. P450-mediated resistance is probably the most frequent type of metabolism based insecticide resistance [44], [45]. This mechanism may potentially affect several classes of insecticides and thereby confer cross-resistance to unrelated compounds due to their broad substrate spectra [46]. Most cases of P450-mediated resistance result from an increase in detoxification. Resistance can occur by increased transcription of a P450 leading to both increased expression of the protein and increased detoxification of the insecticide [47]. In addition to over-expressions of several esterase genes, acephate-selected bugs (LLR) showed increased gene expressions of at least 3 different P450 genes. In the Mississippi Delta area, a variety of insecticides have been used for cotton insect control. Tarnished plant bug has a history of exposure to organophosphates and pyrethroids, and resistances to these pesticides have been reported [9], [5]. In recent years, neonicotinoids have become a popular alternative for plant bug control. P450s are associated with resistance to both pyrethroids [44] and neonicotinoids [48]. Elevated expressions of at least three P450 genes may confer multiple and/or cross resistance to the three commonly used insecticide classes. Our dose-response assays on field populations of TPB (Fig. 6) highly supported this statement of multiple/cross resistance in LLR. Dose-response assays on multiple populations (including Lula) indicated a close correlation between survival rates of TPB treated separately with LC_50_s of acephate and imidacloprid. We are currently trying to develop a P450 enzyme activity assay method to link enzyme activity and elevated P450 gene expression with biological data.

### Down-regulation of eggshell, vitellogenin, protease genes associated with fitness cost

Insect eggshell is composed of a set of proteins (rich in proline and alanine) synthesized by the follicular epithelium during the oogenesis and organized into an inner zone (vitelline membrane) and an outer zone (chorion) [49]. Vitellogenin is a unique group of proteins that are synthesized extraovarially and become the major egg yolk protein, vitellin [50]. Vitellogenin production by females is a prerequisite for successful egg production, and is directly linked to survival and reproductive success, thereby affecting both individual and colony-level fitness [51]. Serine proteases are enzymes that cleave peptide bonds in proteins. They are responsible for coordinating various physiological functions, including digestion, immune response, blood coagulation and reproduction [52]. Very little research has been conducted to link eggshell, vitellogenin, and protease production with fitness cost and resistance to conventional insecticides. We observed a substantial decrease of egg production in survivals after treatment with Orthene 90WP at 240 mg/L. In this study, we report an association of organophosphate resistance with down-regulations of eggshell, vitellogenin, and protease genes in *L. lineolaris*.

Microarray data revealed significant down-regulations of eggshell, vitellogenin, and protease genes in LLR. The finding could be valuable in addressing resistance issues in *L. lineolaris*. When selection pressure increases, either through laboratory selections or field sprays, resistant bugs with elevated esterase and P450 levels could survive and relatively increase resistance gene frequency after removal of susceptible bugs. But, reproductive incompetency substantially limits resistant population growth. When selection pressure is low and suitable host are abundantly available, susceptible bugs, with higher quantities of proteases and eggshell/yolk proteins, take reproductive advantage and increase population density quickly. Considering seasonal fluctuation and exposure-driven natures, we suggest that the acephate resistance in TPB is controlled by multiple genes. We also suggest that the resistance is associated with certain fitness cost.

In summary, microarray gene expression, Blast2go annotation, and other molecular comparisons, in concert with bioassays and enzyme activity data, revealed a significant increase of metabolic processes in the acephate-selected strain, suggesting metabolic detoxification as a major resistance mechanism. The resistance is controlled by many genes. Esterases are critical in detoxification of acephate, and suppression of esterases with inhibitors could significantly abolish acephate resistance. P450s also played an important role, along with less significant influence of GSTs. Down-regulations of many reproductive- and digestive-related genes indicated a potential fitness cost, which might dynamically keep the resistance from becoming fixed in the population. However, up-regulation of P450s and GSTs may increase the risk of multiple and/or cross resistance to other insecticide classes. Precautions must be taken to reduce selection pressure on target insect. Genetic composition of field populations should be constantly monitored to prevent potential genetic shift.
